# Genome-wide DNA methylation analyses in the brain reveal four differentially methylated regions between humans and non-human primates

**DOI:** 10.1186/1471-2148-12-144

**Published:** 2012-08-16

**Authors:** Jinkai Wang, Xiangyu Cao, Yanfeng Zhang, Bing Su

**Affiliations:** 1 State Key Laboratory of Genetic Resources and Evolution, Kunming Institute of Zoology, Chinese Academy of Sciences, 32 East Jiao-Chang Road, Kunming, Yunnan, 650223, People's Republic of China; 2 Graduate School of Chinese Academy of Sciences, Beijing, People's Republic of China

**Keywords:** DNA methylation, Brain evolution, Primates

## Abstract

**Background:**

The highly improved cognitive function is the most significant change in human evolutionary history. Recently, several large-scale studies reported the evolutionary roles of DNA methylation; however, the role of DNA methylation on brain evolution is largely unknown.

**Results:**

To test if DNA methylation has contributed to the evolution of human brain, with the use of MeDIP-Chip and SEQUENOM MassARRAY, we conducted a genome-wide analysis to identify differentially methylated regions (DMRs) in the brain between humans and rhesus macaques. We first identified a total of 150 candidate DMRs by the MeDIP-Chip method, among which 4 DMRs were confirmed by the MassARRAY analysis. All 4 DMRs are within or close to the CpG islands, and a MIR3 repeat element was identified in one DMR, but no repeat sequence was observed in the other 3 DMRs. For the 4 DMR genes, their proteins tend to be conserved and two genes have neural related functions. Bisulfite sequencing and phylogenetic comparison among human, chimpanzee, rhesus macaque and rat suggested several regions of lineage specific DNA methylation, including a human specific hypomethylated region in the promoter of *K6IRS2* gene.

**Conclusions:**

Our study provides a new angle of studying human brain evolution and understanding the evolutionary role of DNA methylation in the central nervous system. The results suggest that the patterns of DNA methylation in the brain are in general similar between humans and non-human primates, and only a few DMRs were identified.

## Background

The most significant difference between humans and non-human primates is the highly developed cognitive ability in humans. Though many efforts have been made to delineate the genetic basis of the cognitive difference, it remains unclear. Because of the great similarity of genome sequences between humans and non-human primates [1,2], more and more studies have started to look at gene regulation differences in the brain [3,4], which are thought to be much more important for human brain evolution. As epigenetic modifications play important roles in learning and memory [5-7], it is possible that epigenetic modifications have contributed to human brain evolution. However, this has rarely been addressed.

Comparison of DNA methylation between humans and non-human primates at the orthologous regions in the genome could be an informative attempt to reveal the evolutionary roles of DNA methylation in human brain evolution. Two small-scale studies that compared DNA methylation of brain between humans and non-human primates have been conducted. It was reported that DNA methylation differences between human and chimpanzee were predominantly observed in the brain [8]. Another study reported a species-specific methylated region in an Alu-Sg1 repeat [9]. Recently, several large-scale studies of DNA methylation comparison between humans and non-human primates in multiple tissues (brain was not included) and cell lines have also been conducted, and the differentially methylated regions were identified mostly in repeats and CpG islands [10-12]. But the situation of DNA methylation in the brain, which is intuitively more important for primates, is largely unknown.

We aim to identify genes that show differential DNA methylation levels in the prefrontal cortex (PFC) between humans and non-human primates. We first conducted a genome-wide methylation scan using the MeDIP-Chip technology, and then the candidate regions were subject to the analysis using SEQUENOM MassARRAY [13] for confirmation and further validation using independent samples. Finally, we identified four regions/genes differentially methylated between humans and rhesus macaques in PFC. Two genes have neural related functions. The phylogenetic comparison among human, chimpanzee, rhesus macaque and rat suggested that a DMR in the promoter of *K6IRS2* was hypomethylated specifically in the human brain, not the other species.

## Results

### General profile of genome-wide DNA methylation

Firstly, three adult human and three age/sex matched rhesus macaque (Table 1) PFC samples were subjected to the MeDIP-Chip analysis to obtain the genome-wide DNA methylation data. For rhesus macaques, the species-specific arrays were designed and used (see Materials and Methods for details).

**Table 1:** Information of samples

**Sample ID**	**Species**	**Sex**	**Age (years)**	**Cortex Region**
H1^a,d^	Human	Male	40	PFC^c^
H2^a^	Human	Male	28	PFC^c^
H3^a^	Human	Male	59	PFC^c^
H4^b^	Human	Female	35	PFC^c^
H5^b^	Human	Male	7.5	PFC^c^
M1^a,d^	Rhesus macaque	Male	13	PFC^c^
M2^a^	Rhesus macaque	Male	9	PFC^c^
M3^a^	Rhesus macaque	Male	19	PFC^c^
M4^b^	Rhesus macaque	Male	16	PFC^c^
M5^b^	Rhesus macaque	Male	1-2	PFC^c^
M6^b^	Rhesus macaque	Male	11	PFC^c^
M7^b^	Rhesus macaque	Female	13	PFC^c^
Chimpanzee1^d^	Chimpanzee	Male	1.5	PFC^c^
Chimpanzee2^d^	Chimpanzee	Male	1.5	PFC^c^
Rat1^d^	Rat	Female	2 months	PFC^c^
Rat2^d^	Rat	Male	2 months	PFC^c^

^a^the 6 samples for MeDIP-Chip and MassARRAY analyses.

^b^additional independent samples used for further validation.

^c^prefrontal cortex.

^d^the samples used for multiple species comparison using bisulfite clone sequencing.

**Figure 1:**
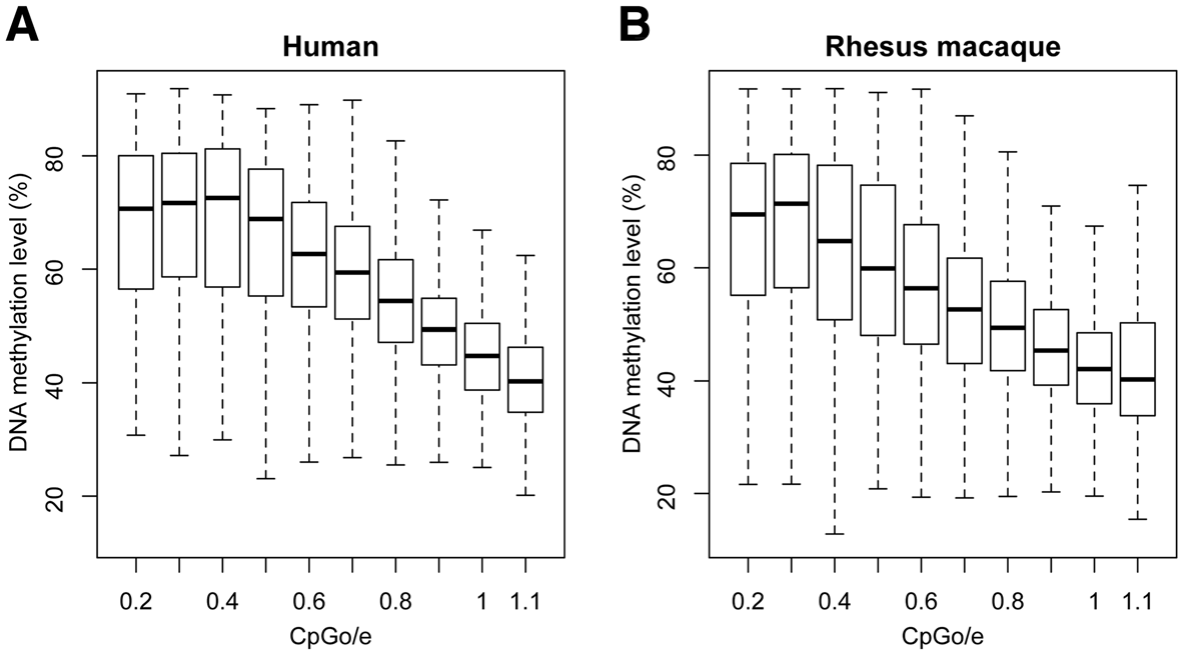
** Negative correlation between DNA methylation and CpGo/e ratio (the observed vs. the expected CpG ratios) in humans (A) and rhesus macaques (B).** For each 2700 bp promoter region, the 540bp window closest to the transcription start site was used. The regions were grouped into bins based on their CpGo/e ratios, and at least 40 regions were required in each bin. The X-axis label of each bin represents the minimum CpGo/e value. For example, “0.2” refers to a bin with the CpGo/e values ranging between 0.2 and 0.3. The methylation levels were the average of 3 individual samples in each species.

For the MeDIP-Chip data, we first tested the correlation between CpG content and DNA methylation level. For each 2,700 bp promoter region, we only used the 540bp window closest to the transcription start site, and we observed a significant negative correlation (in humans, Spearman R = −0.546, P < 2.2e-16; in rhesus macaques, Spearman R = −0.500, P < 2.2e-16) (Figure 1). This is consistent with previous studies showing that in mammalian species, CpG islands, which have higher CpGo/e, usually have lower methylation levels [14,15].

We then tested if the DNA methylation level was negatively correlated with gene expression using the published gene expression data [16]. As the coverage of the expression data of rhesus macaques was not high enough, we only tested the correlation in humans. We used a 540 bp sliding window to access the correlations between gene expression and the methylation levels of different locations of the 2,700bp promoter regions. As shown in Additional file 1 (Figure S1), all the five sliding windows showed significant negative correlation. Interestingly, the region of -40bp-500bp showed a non-monotonic relationship, which is consistent with the recent finding that the gene-body methylation has a non-monotonic relationship with gene expression [17].

### Identification of candidate regions showing differential DNA methylation

**Figure 2:**
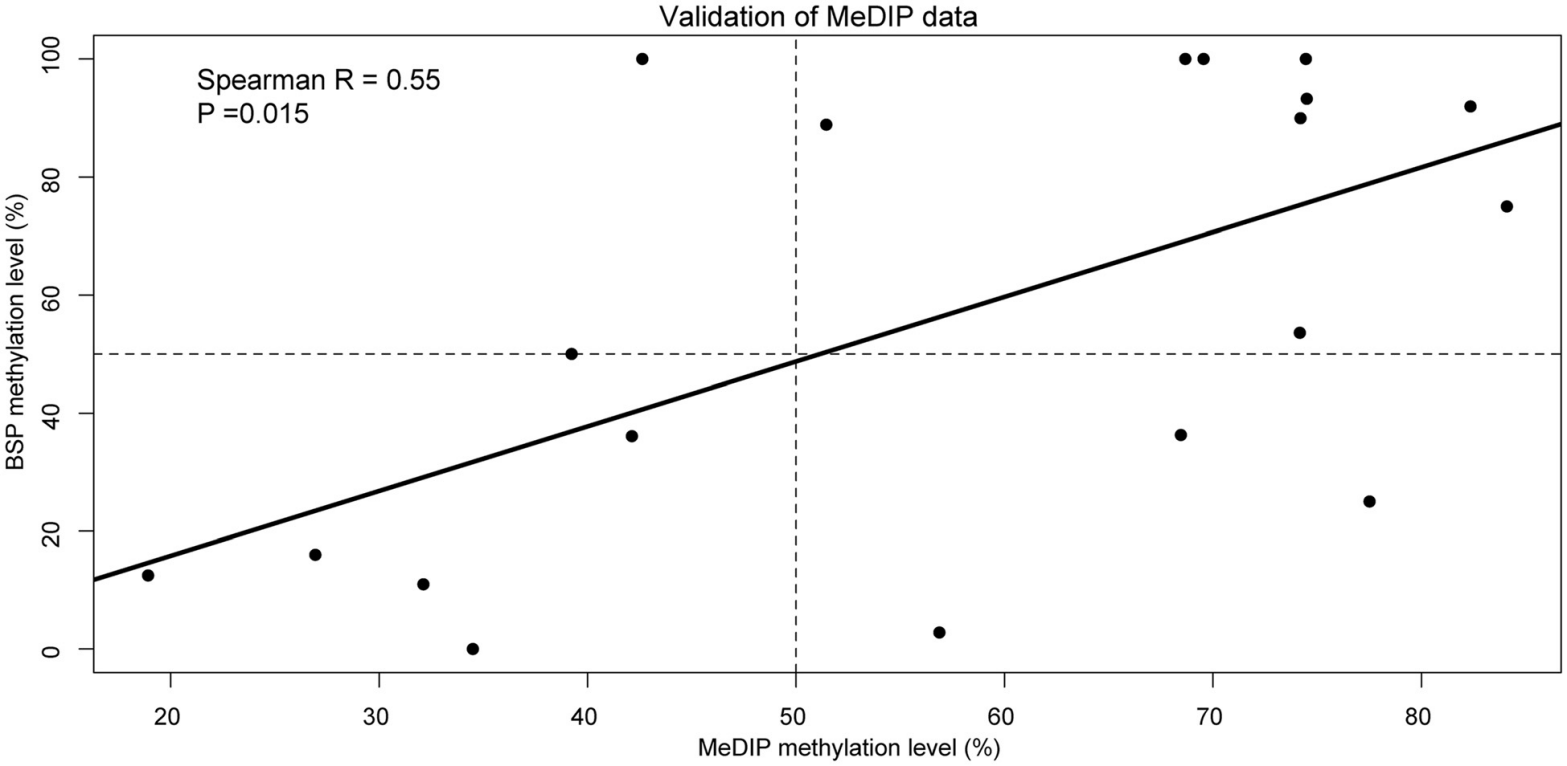
Correlation between the methylation levels of MeDIP-Chip and bisulfite clone sequencing (BSP).

To check the quality of the MeDIP-Chip data, we randomly selected 19 regions with different DNA methylation levels and CpG contents and conducted bisulfite sequencing. The result showed that there was a good correlation between the MeDIP-Chip data and the bisulfite sequencing data (Spearman R = 0.55, P = 0.015) (Figure 2), indicating that the MeDIP-Chip data was informative and relatively reliable as a preliminary screening.

To identify the candidate differentially methylated regions between humans and rhesus macaques, we used two different methods to analyze the MeDIP-Chip data: the NimbleScan and the Batman methods [18]. The regions that were estimated having differential DNA methylation levels by both methods were selected as the candidate regions for further analysis. Around 42% of the peaks identified by NimbleScan (*i.e.* the peaks only observed in either of the two species) were confirmed by Batman (Additional file 2). As a preliminary screening step, we used a nominal P value cutoff of 0.05 to select the candidate regions. Totally, we obtained 150 candidate DMRs (Additional file 3), and most of them were located (or partly located) in the CpG islands (142 for humans and 141 for rhesus macaques) based on the UCSC genome annotation (http://genome.ucsc.edu/). This is consistent with the features of the MeDIP-Chip technology, which tends to find CpG methylation in the high CpG density regions [19]. Gene ontology (GO) analysis for the 150 candidate DMRs was conducted using DAVID[20], however, no enrichment was observed.

It has been suggested that there is a high false discovery rate for the MeDIP-Chip method [21]. And even the DMRs identified between two human embryonic stem cell lines using the more advanced MeDIP-seq technique do not have good validation [22]. Hence, the 150 candidate DMRs need to be further tested by independent methods.

### Validation of the candidate DMRs using MassARRAY

For the 150 candidate regions likely having differential DNA methylation levels between humans and rhesus macaques, we conducted further analysis in the same samples (3 human versus 3 rhesus samples) using the Sequenom MassARRAY method [13], which has much higher resolution and accuracy for DNA methylation compared to the MeDIP-Chip method and is suitable for candidate gene validation. Among the 150 candidate DMRs, a total of 118 DMRs were successfully amplified and obtained eligible data for more than one sample in both species. It should be noted that for the MassARRAY method, the minimum measuring unit was CpG unit, which are one or several consecutive CpG sites. A total of 948 eligible CpG units were obtained for the 118 DMRs, and 688 of them can be paired based on their orthology between humans and rhesus macaques. The other 260 eligible CpG units could not be paired, therefore, were excluded from further analyses.

**Figure 3:**
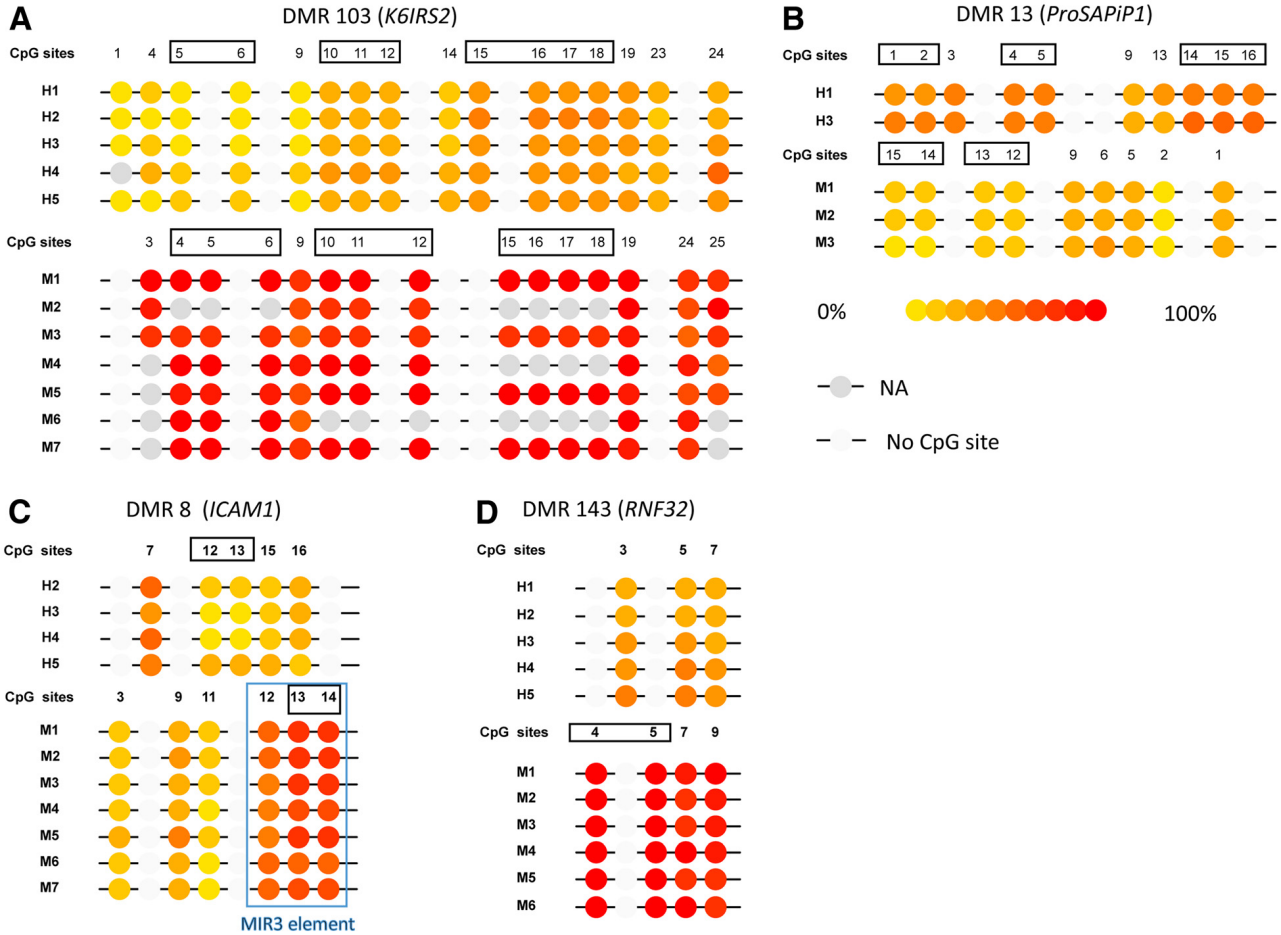
** The illustration of the DMRs between humans and rhesus macaques from the Sequenom MassArray data.** The CpG sites in the black rectangle are in a single CpG unit, and the methylation levels of each CpG site of the CpG unit is represented by the methylation level of that CpG unit. The CpG sites are numbered from 5' to 3' of the sequences of the PCR products on the measured DNA strands (Additional file 3). The CpG units that cannot be paired and not used in the study due to lacking of orthologous CpG units in the counterpart species are also shown in this figure. The 4 DMRs validated by the MassARRAY method are shown in **A**-**D** respectively. The region in blue rectangle is the MIR3 repeat element.

For each of the 118 candidate DMRs, the average methylation level of each region was calculated and compared between humans and rhesus macaques. Two criteria were used to determine the validated DMRs: (1) the differences of the average methylation levels between the two species should be larger than 0.2 as reported previously in order to reduce false discovery [23]; (2) the p value adjusted by Benjamini FDR should be less than 0.05. Using these statistical criteria followed by bisulfite sequencing (data not show), we identified a total of 4 DMRs (Figure 3, Table 2). The directions (increased or decreased DNA methylation levels) of the differences between humans and rhesus macaques for all 4 DMRs were the same as that of the MeDIP-Chip results, suggesting that the initial screening using MeDIP-Chip was effective though not accurate. Notably, since we used relatively loose criteria to select the candidate DMRs from the MeDIP-Chip data in order to cover more potentially differentially methylated regions. Thus, it was not surprising that only a small part of the 150 candidate DMRs were validated by the MassARRAY analysis.

**Table 2:** The four DMRs (differentially methylated regions) validated by the MassARRAY analysis

**DMR ID**^**a**^	**Gene**	**Human PCR region**^**b**^	**Rhesus PCR region**	**H1**	**H2**	**H3**	**M1**	**M2**	**M3**	**Differences**	**P value**^**c**^
143	*RNF32*	chr7:156125452-156125599	chr3:193770671-193770806	0.287	0.260	0.310	0.953	0.903	0.905	−0.647	8.2E-06
103	*K6IRS2*	chr12:51281338-51281660	chr11:49678948-49679270	0.225	0.235	0.242	0.951	0.801	0.738	−0.596	7.2E-04
8	*ICAM1*	chr19:10242793-10243123	chr19:10081432-10081787	0.148	0.145	0.160	0.540	0.540	0.530	−0.383	2.8E-07
13	*ProSAPiP1*	chr20:3096748-3097085	chr10:36475835-36476155	0.400	0.355	0.446	0.183	0.170	0.130	0.239	1.5E-03

^a^the ID number of the 150 candidate DMRs identified by MeDIP-Chip.

^b^the coordinates are based on hg18 build.

^c^the P values obtained by two-sided *t*-test, and all P values are smaller than 0.05 after Benjamini FDR adjustments.

To further confirm the 4 DMRs, using MassARRAY, we tested an independent sample group (2 humans vs. 4 rhesus macaques). As shown in Table 3 and Figure 3, 3 of the 4 DMRs were successfully replicated, and the other DMR failed to be confirmed due to low data quality. When all the samples were analyzed together, the 4 DMRs remained significant (Table 3).

**Table 3:** Validation of DMRs in additional independent samples

**DMR ID**	**Gene**	**H4**	**H5**	**M4**	**M5**	**M6**	**M7**	**Differences**	**P value**	**Corrected P value**^**a**^	**P value (all)**^**b**^
103	*K6IRS2*	0.289	0.252	0.936	0.852	0.902	0.958	−0.641	6.22E-05	1.9E-04	1.7E-08
143	*RNF32*	0.403	0.427	0.930	0.875	0.925	NA^c^	−0.495	2.6E-04	7.8E-04	2.3E-08
8	*ICAM1*	0.103	0.200	0.455	0.520	0.410	0.500	−0.320	2.5E-03	7.5E-03	9.7E-08

^a^the P values after Bonferroni correction by 3 tests.

^b^the nominal P values obtained using all the samples including both MeDIP-Chip samples and additional independent samples.

^c^not available.

In the 4 validated DMRs, there are 25 CpG units that can be paired and studied. As shown in Figure 3, not all the CpG units in the 4DMRs show different methylation levels between humans and rhesus macaques, *e.g.* DMR8. To look into the details of the methylation difference, we compared the DNA methylation levels of single CpG unit for the 688 CpG units that could be paired and used in the analysis. And we identified 25 pairs of CpG units that showed significant differential DNA methylation levels, in which 14 pairs of CpG units were from the 4 validated DMRs (Table 4, Figure 3).

**Table 4:** Differentially methylated CpG units between humans and rhesus macaques

**DMR ID**	**Human CpGunit**^**a,c**^	**Rhesus CpGunit**^**b,c**^	**H1**^**d**^	**H2**^**d**^	**H3**^**d**^	**M1**^**d**^	**M2**^**d**^	**M3**^**d**^	**Differences**	**P value**^**e**^	**Corrected P value**^**f**^
8	16 NA	13 14	0.26	0.24	0.25	0.73	0.72	0.73	−0.48	2.3E-07	7.9E-05
8	15	12	0.17	0.12	0.19	0.55	0.58	0.49	−0.38	3.5E-04	8.6E-03
13	NA 4 5	13 12 NA	0.41	NA	0.41	0.17	0.12	0.14	0.27	7.5E-04	1.3E-02
13	14 15 16	NA 1 NA	0.49	NA	0.57	0.29	0.3	0.28	0.24	4.4E-03	4.0E-02
13	1 2	15 14	0.37	0.44	0.48	0.17	0.17	0.02	0.32	6.1E-03	5.0E-02
22	7 8 9 10	7 8 9 10	0.23	0.23	0.12	0.43	0.48	0.41	−0.25	4.3E-03	4.0E-02
51	17 18 19	16 17 18	0.07	0.02	0.05	NA	0.27	0.3	−0.24	1.6E-03	2.2E-02
57	9 NA 10 11	8 9 10 NA	0.11	0.13	0.08	0.67	NA	0.66	−0.56	8.8E-05	5.0E-03
57	29 NA	25 26	0	0	0	0.18	NA	0.25	−0.22	3.7E-03	3.8E-02
75	NA 1	1 2	0.76	0.75	0.8	1.00	1.00	1.00	−0.23	1.1E-04	5.1E-03
76	26 27	NA 1	1.00	1.00	1.00	0.62	0.59	0.69	0.37	2.4E-04	7.3E-03
103	9	9	0.01	0.03	0.03	0.77	0.69	0.6	−0.66	1.8E-04	6.5E-03
103	19	19	0.38	0.33	0.38	0.97	0.91	0.81	−0.53	4.2E-04	9.7E-03
103	4	3	0.14	0.07	0.13	1.00	0.81	0.75	−0.74	7.0E-04	1.3E-02
103	10 11 12 NA	10 11 NA 12	0.21	0.22	0.21	0.99	0.74	0.76	−0.62	1.5E-03	2.1E-02
103	5 NA 6 NA	4 5 NA 6	0.05	0.04	0.06	0.96	NA	0.74	−0.81	2.3E-03	2.6E-02
103	24	25	0.24	0.26	0.31	0.73	0.98	0.71	−0.54	3.9E-03	3.9E-02
122	3 4 5	NA 5 NA	0.87	0.77	0.87	0.26	0.26	0.24	0.58	6.8E-05	5.0E-03
127	18 19 NA	18 19 20	0.11	0.04	0.10	0.28	0.3	0.30	−0.21	7.8E-04	1.3E-02
131	43 44	31 32	0.21	0.24	0.27	0	0.03	0.01	0.23	3.1E-04	7.9E-03
139	15 16 NA 17 18 19	8 9 10 NA 11 NA	0.13	0.21	0.17	0.36	0.39	0.41	−0.22	1.4E-03	2.0E-02
141	45 46 47	43 NA 44	0.25	0.20	0.20	0.02	0.01	0.01	0.21	2.9E-04	7.9E-03
143	NA 3 NA	4 NA 5	0.30	0.29	0.35	1.00	1.00	0.99	−0.68	3.5E-06	7.9E-04
143	7	9	0.27	0.22	0.25	0.96	0.86	0.84	−0.64	8.8E-05	5.0E-03
143	5	7	0.29	0.27	0.33	0.85	0.75	0.80	−0.50	1.2E-04	5.1E-03

^a^the CpG sites which constitute the CpG unit of human.

^b^the CpG sites which constitute the CpG unit of rhesus macaque.

^c^the orthologous CpG sites between human CpG units and rhesus macaque CpG units are in the same order; NA means no CpG site in this species but a CpG site in the counterpart species at the orthologous site.

^d^NA means not available.

^e^the P values were obtained by two-sided *t*-test. Corrected P value.

^f^the P value that corrected by Benjamini FDR.

### Features and functional implication of the DMRs

For the 4 DMRs, DMR143 is located in the promoter of *RNF32*; DMR103 covers the promoter and the first exon of *K6iRS2*; DMR8 covers the first exon and extends to the first intron of *ICAM1*, and DMR13 is located in the first exon of *ProSAPiP1.* Based on the UCSC human genome annotation, DMR143 is entirely located in the CpG island; DMR103 and DMR8 have more than half of their full length located in the CpG islands, and DMR13 is located 2,992bp from the nearest CpG island.

We then checked if there were any repeat elements in the DMRs. We found nearly all the 4 DMRs had no repeat element, because we removed the non-unique probes of MeDIP-Chip array, which would remove most of probes targeted to repeat elements. However, there was an exception in rhesus macaques where we found a MIR3 repeat element that was entirely located in DMR8 (*ICAM1* gene) based on the UCSC genome annotation.

To look into the potential functional significance of the differentially methylated regions and its implications for human evolution, we conducted functional annotation for the four genes located in the 4DMRs. Interestingly, two genes are involved in neural functions. *ICAM1* (*intercellular adhesion molecule 1*) is a transmembrane glycoprotein, which belongs to the immunoglobulin superfamily, and has been reported associated with schizophrenia [24] and Alzheimer’s disease [25]. *ProSAPiP1* is a synaptic protein that interacts with the PDZ domain of *ProSAP2*/*Shank3*[26]. For the other two genes, *K6IRS2* encodes a type II keratin that is expressed in the inner root sheath of hair follicles [27], and the function of *K6IRS2* is not known. *RNF32* is a ring finger protein, and its function is unknown.

Next, we addressed the functional conservation of the 4 DMRs genes, and we studied their protein conservation using human versus rhesus macaque dN/dS ratios (ratio of nonsynonymous substitution rate vs. synonymous substitution rate). The dN/dS ratios of all 4 genes were smaller than one, suggesting functional conservation (0.14 for *RNF32*, 0.16 for *K6IRS2*, 0.13 for *ProSAPiP1* and 0.49 for *ICAM1*). These results suggest that the identified DMRs are not likely caused by protein functional divergence between different species.

### DNA methylation comparison in multiple species

**Figure 4:**
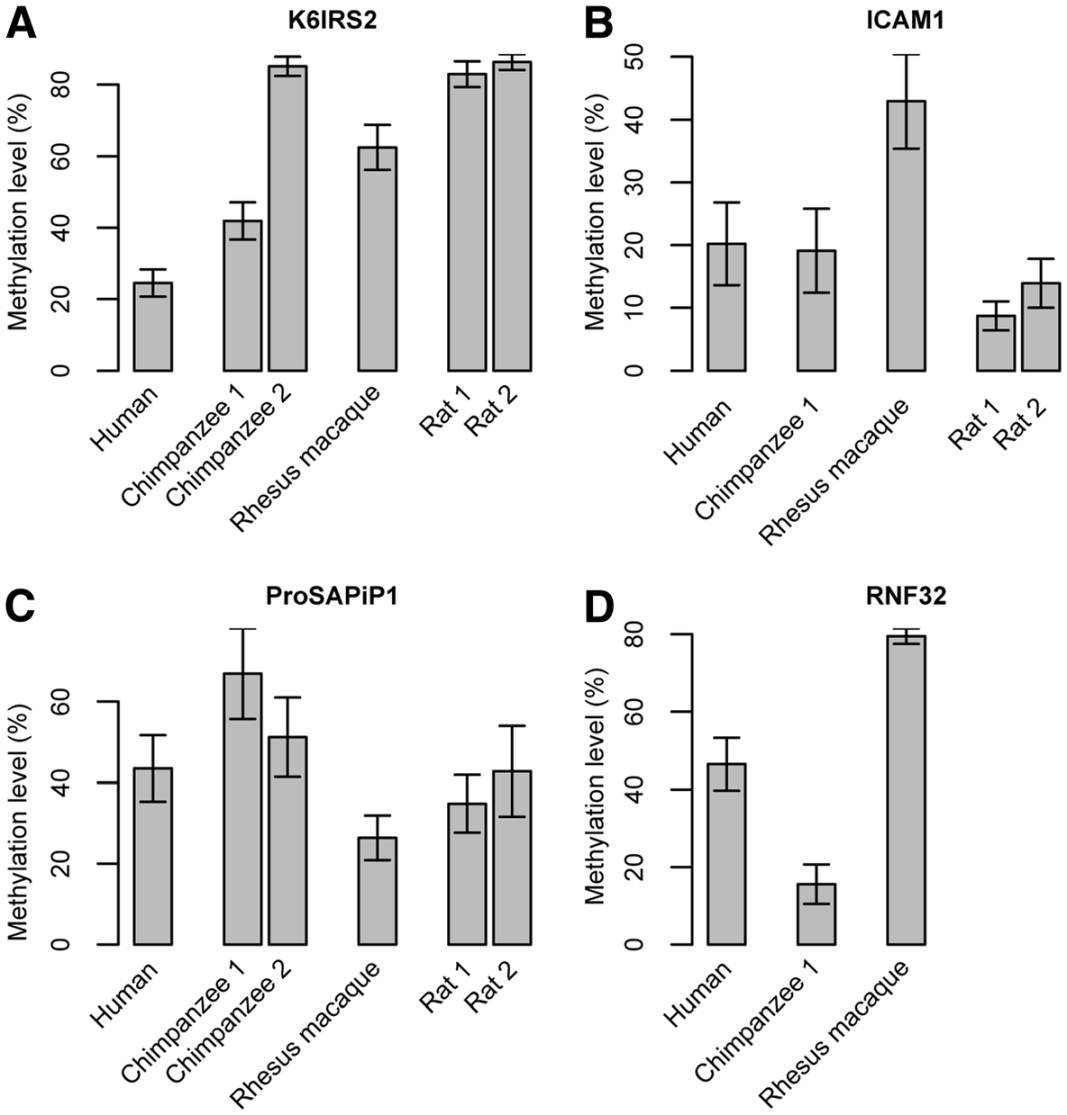
** Comparisons of DNA methylation of 4 DMRs in PFC of multiple species.** For each DMR, the orthologous regions (annotated by UCSC pairwise genome alignment) of 4 species were compared. DNA methylation levels were obtained by bisulfite clone sequencing, and the comparisons for each DMR are shown in **A**-**D** respectively. There were missing of Chimpanzee2 due to failed PCRs. The sequenced samples for human and rhesus macaque were H1 and M1. For RNF32 (**D**), there is no orthologous gene in the rat genome. The error bars represent the standard errors of the methylation levels of all the CpG sites of a gene.

To look into the detailed evolutionary changes of the identified DMRs, using bisulfite sequencing, we measured the DNA methylation levels of the 4 DMRs in multiple species including human, chimpanzee, rhesus macaque and rat. The methylation levels of the orthologous regions of the DMRs were compared in the phylogenetic context with rat as outgroup. Interestingly, the four DMRs showed different patterns. For *ICAM1*, chimpanzee and rat showed similar methylation levels with humans, while rhesus macaque had a lineage specific increase of methylation level (Figure 4B). Similar pattern was observed for *ProSAPiP1* where rhesus macaque had a lineage specific decrease of methylation (Figure 4C). For *RNF32*, the methylation levels were different among human, chimpanzee and rhesus macaque and there is no orthologous gene in rat (Figure 4D). The most informative case was *K61RS2*, in which the methylation level in humans showed a lineage-specific decrease (hypomethylation) (Figure 4A), suggesting a human-specific methylation change during evolution.

## Discussion

In this study, we employed a two-step method to identify the differentially methylated regions and 4 regions were found to have different methylation levels in PFC between humans and rhesus macaques, which are informative candidates for further study of human brain evolution. However, there are also methodological limitations. The 150 candidate regions are all high CpG content regions because the NimbleGen method tends to find highly methylated regions with high CpG content. It was reported that high CpG content regions were of higher quality in MeDIP-Chip data [21]. In studies of tissue specific DNA methylation regions, high CpG content regions such as the CpG islands showed less between individual variance and less differences between different tissues [28-30], and this may be the same case between different species. Though we cannot exclude the possibility that there are differentially methylated regions in low CpG content regions, the high CpG content regions, on which we focused in this study, are more likely to be functionally important [14,15] and more likely to have definite between-species differences and less within-species variations, thus evolutionarily more significant. In addition, considering the multiple data filtering steps using different methods, the number of identified DMRs in this study was likely an underestimation of the between-species methylation divergence in the brain.

Repeat elements are important in DNA methylation. Farcas *et al.*[9] reported a small region of ALU-Sg1 element that was differentially methylated between human and chimpanzee cerebral cortex. In this study, we found another case of SINE element differentially methylated between humans and rhesus macaques, implying the roles of repeat elements in the epigenetic evolution of primates. In the rhesus macaque genome, the MIR3 element located in DMR8 has a length of 98 bp and divergence of 34.4% to its consensus sequence. In DMR8, the human ortholog of this region have 6 substitutions and 1 deletion compared with rhesus macaque, and it is not annotated as repeat region by UCSC. MIR3 belongs to the MIR (Mammalian-wide interspersed repeats) family, which is one of the oldest tRNA-derived SINE (short interspersed element) elements, and they integrated into the host genomes before the radiation of mammals [31]. In DMR8, three CpG sites (CpG12, CpG13 and CpG14) of rhesus macaques are located in MIR3. Interestingly, the three CpG sites are the only CpG sites that showed significant methylation differences between humans and rhesus macaques (Figure 3 and Table 4), suggesting that the MIR3 element has led to the differential methylation of DMR8 between humans and rhesus macaques.

Among the 4 validated DMRs, only one is located in the repeat region, suggesting that the differentially methylated regions between humans and nonhuman primates are not necessarily restricted to repeat regions and can be high CpG content regions, which have been previously thought to be less variable [28-30].

As DNA methylation in the promoter regions will repress gene expression, it would be informative to see whether the differential DNA methylations between humans and rhesus macaques would lead to expression differences. We analyzed the published gene expression data from human and rhesus macaque cerebral cortices [16]. Among the four DMRs identified in our study, two DMR related genes (*ICAM1*and*ProSAPiP1*) had eligible expression data and showed significant gene expression differences between human and rhesus macaque (P = 0.000757 and 0.00764 for *ICAM1* and *ProSAPiP1* respectively). However, for both genes, the species having higher DNA methylation levels also showed higher gene expression levels, which is inconsistent with the expected repression of gene expression by a higher level of DNA methylation in the promoter. However, these two DMRs also covered the first exons of the corresponding genes, and the gene-body methylation was reported to be positively correlated with gene expression in human cells [32]. Hence, detailed analysis in the future is needed to reveal the influence of DMRs on gene expression.

It is difficult to address how the observed methylation differences have been formed during primate evolution. Trans-generational inheritance is crucial if epigenetic modification should play a role in evolution. Many studies have reported that epigenetic modifications can be inherited across generations [33-35]. But the inheritance of DNA methylation is not as stable as that of DNA sequence and the mechanisms underlying the trans-generational inheritance of epigenetic modification are not well understood.

Recently, it was reported that DNA methylation is correlated with DNA sequence variations [36-38] and non-coding RNAs [39-42], and these correlations might act as an indirect mechanism that explains the trans-generational inheritance of epigenetic modification. The DNA sequence substitutions during evolution may cause the emergence of new CpG sites and/or loss of the existing CpG sites, eventually leading to methylation divergence of specific genomic regions. It has been reported that the CpG-SNPs in the human genome have contributions to allele specific DNA methylation [43], implying that even at the population level, the methylation divergence can occur due to DNA sequence polymorphisms. Alternatively, DNA sequence differences could also result from DNA methylation differences between species [10-12].

Another possibility is that epigenetic modifications altered by environment can be transmitted to the next generation directly. Global epigenetic reprogramming including demethylation of DNA occurs in the mammalian primordial germ cells and in early embryos [34]. Although global epigenetic reprogramming will restrict the trans-generational epigenetic inheritance, the erasure of DNA methylation modifications is not absolute [44,45], suggesting the possibility of direct transmitting of DNA methylation modifications to the next generation. Additionally, at the whole genome level, the over-all similar DNA methylation patterns between humans and macaques cannot be explained by the similarity of living environment because they inhabit totally different environments, suggesting potentially vertical inheritance of methylation.

A lot of candidate DMRs obtained from the MeDIP-Chip analysis were not validated by MassARRAY and bisulfite sequencing. This discrepancy could be explained by two possible sources. First, for MeDIP-Chip analysis, we used different arrays for humans and rhesus macaques. Even though the validation for each species-specific array is good, the technical bias may still exist, as the array for rhesus macaques has not been rigorously tested. Second, besides detecting the genome-wide DNA methylation patterns, we focused on identifying differentially methylated regions between humans and non-human primates. Thus, a relaxed cut-off (nominal P smaller than 0.05 without multiple test correction) was applied for selecting the 150 candidates from the MeDIP-Chip data, likely resulting in a relatively high number of false-positives.

## Conclusion

Our study is the first large-scale comparison of DNA methylation between human and non-human primates in the brain. We found several regions of lineage specific DNA methylation, indicating that DNA methylation plays potential roles in brain evolution, and the regions we reported are good candidates for further functional studies, and will shed light on the study of human brain evolution. The results suggest that the patterns of DNA methylation in the brain are in general similar between humans and non-human primates.

## Methods

### Tissue samples

Frozen tissues were obtained from PFC of 5 humans, 7 rhesus macaques, two chimpanzees and two rats with no known neuronal diseases or drug abuse. Five of the 7 rhesus macaques and all 5 human samples were sex and age matched, following the reported criteria that the ages of humans are in general 3 times of the ages of rhesus macaques [46]. These age-matched human and macaque samples (3 from each species) were used in the initial genome-wide scanning using the MeDIP-Chip method. The information of the samples is shown in Table 1. For the human subjects, informed consents were obtained from their relatives. The use of animal tissues follows internationally recognized guidelines. The research protocol was approved by the internal review board of Kunming Institute of Zoology, Chinese Academy of Sciences (reference number: SWYX-2010-002).

### MeDIP-Chip (Methylated DNA immunoprecipitation)

Genomic DNA from PFC was extracted using DNeasy Blood & Tissue Kit (Qiagen) and fragmented by ultrasonic method following the manufacturer’s instructions. Immunoprecipitation of methylated DNA was performed using anti-5-methyl cytidine (mouse) and BiomagTM magnetic beads coupled anti-mouse IgG. Immunoprecipitated DNA was eluted and purified by phenol chloroform extraction. Then the Input and IP DNAs were labeled with Cy5- and Cy3-labeled random 9-mers respectively. The labeled human and rhesus macaque DNAs (Input and IP) were hybridized to NimbleGen HG18 RefSeq promoter arrays and the customer designed rhesus macaque DNA methylation array respectively. The scanning was performed with the Axon GenePix 4000B microarray scanner.

The NimbleGen HG18 RefSeq promoter array is a single array design containing all known well-characterized 18,028 RefSeq promoter regions (from about -2200bp to +500bp of the transcription start sites) totally covered by about 385,000 probes. The customer-designed rhesus macaque array was designed according to the commercial NimbleGen HG18 RefSeq promoter array. The regions covered on HG18 RefSeq promoter array were aligned to the rhesus macaque genome using BLAT software, and the alignments that have more than two hits were removed. Then the aligned rhesus macaque regions were merged if they overlapped, regions shorter than 1,700bp were removed because these regions were likely the false orthologous regions or those with large sequence divergence between the two species. The regions shorter than its corresponding human regions were extend equally on both sides to make the regions of both species equal length. Then the probes of rhesus macaque array were designed and synthesized according to these genomic regions. In all the aligned regions, only the Ensembl annotated one to one orthologous gene pairs were subjected to the following DNA methylation and comparison between humans and rhesus macaques.

### MeDIP-Chip data analyses

The raw data were extracted by NimbleScan software. In the analyses of the genome-wide profiling of MeDIP-Chip data, the methylation levels of every sliding window of 540bp were calculated using Batman software [18]. Before the analysis, the quantile normalization has been conducted using Limma package [47] implemented in R, and the probes that are not unique in the respective genomes were removed from the analysis.

Two different methods were used to obtain the list of candidate DMRs from the MeDIP-Chip data in order to reduce the false discovery rate. In the first method, we used the NimbleScan software developed by NimbleGen under its standard procedure and instruction to find the candidate DMRs. The regions were defined to be candidate DMRs only if they were reported methylated in all three samples of one species and none in the other species. Then, the quantitative methylation levels were calculated using Batman and compared between the two species for the regions that were reported to show different methylation status by NimbleScan method, and the differentially methylated regions showed large differences of CpG numbers between the two species were removed as they were likely due to the technical bias of NimbleScan method, which did not use CpG density to do the adjustment. Custom Perl scripts were used to accomplish the above procedures, and the statistics were conducted using R.

The MeDIP-Chip data for the human and rhesus macaque PFC samples can be downloaded from NCBI’s GEO (http:/http://www.ncbi.nlm.nih.gov/geo/) database (accession number: GSE27461).

### Bisulfite sequencing

We used the EpiTect Bisulfite Kits (Qiagen) to conduct the bisulfite conversion of DNA under the manufacturer's instructions, and the PCR primers were designed using Methyl Primer Express (v1.0, ABi Corp.). At least 10 clones were sequenced for each fragment by ABI 3130 sequencer after PCR, ligation and cloning. The clone sequences were then analyzed using BiQ Analyzer software [48] to get the methylation levels after manual checking.

### SEQUENOM MassARRAY experiments and data analyses

Experiments were conducted under manufacturer’s instructions. Because of the difficulty of designing perfect eligible PCR primers with no CpG, for some regions, we allowed one CpG in the primers, and in rare cases, two CpGs were also allowed, and the CpGs in the primers had been removed from data analyses. As for the 4 DMRs found by MassARRAY method, none of them had CpG in its primers. For some regions that no eligible PCR primer could be designed in at least one species, we tested the proximal regions of both species instead.

Any unreliable CpG units (one or more CpG sites that measured as one unit in SEQUENOM MassARRAY technique) reported by EpiTYPER software (SEQUENOM) were removed from our analyses. To compare the methylation levels between humans and rhesus macaques, we used Clustal W software to align the bisulfite-converted sequences of the corresponding human and rhesus macaque regions. Then the orthologous pairs of CpG units of these two species were determined and compared respectively. The orthologous CpG unit pair was defined as the CpG unit pair that had at least one orthologous CpG site and all other CpG sites that did not have orthologous CpG site were due to the lack of CpG site on the counterpart species. Therefore, in some occasions, multiple short CpG units need to be merged in order to pair the longer CpG unit in the counterpart species. To calculate the average DNA methylation level of each region, we used only the CpG units that had corresponding orthologous CpG units in the counterpart species, but the methylation levels of the CpG units without orthologous CpG units were also shown (Figure 3).

The dN/dS values of the 4 DMR genes were obtained from the Ensembl database (http://www.ensembl.org/).

### Gene expression analyses

To analyze the relationship between gene expression and DNA methylation and compare gene expression differences between human and rhesus macaque, we used the published gene expression data [16], and the human and rhesus macaque cerebral cortex samples with similar ages compared to our samples were used (NCBI’s GEO accession number: GSM289929, GSM289938, GSM289944, GSM289947, GSM289948, GSM289950, GSM289951, GSM289972, GSM289973, GSM289974, GSM289975, GSM289976 and GSM289977).

## Competing interests

All authors declare that they have no competing interests.

## Authors’ contributions

Bing Su and Jinkai Wang designed the experiment; Jinkai Wang and Xiangyu Cao performed the molecular biology experiments and data analyses; Jinkai Wang performed bioinformatic analyses; Jinkai Wang and Yanfeng Zhang performed data illustration; Jinkai Wang and Bing Su wrote the manuscript and all authors read and approved the final manuscript.

## Supplementary Material

Additional file 1 **Figure S1.** Relationship between DNA methylation in regions of different distances to transcriptional start sites and gene expression levels in the human cerebral cortex. Click here for file

Additional file 2 **Table S1.** The numbers of DMRs identified by the Batman method when analyzing the 450 peaks identified in either of the two species by the NimbleScan method. Click here for file

Additional file 3 **Table S2.** Information and primers of the 150 candidate regions. Click here for file
