# Supplementary material for: Genome-wide DNA methylation analyses in the brain reveal four differentially methylated regions between humans and non-human primates
Source: BMC Evol Biol. 2012 Aug 16;12:144. doi: 10.1186/1471-2148-12-144 (PMC3483258; doi:10.1186/1471-2148-12-144)
Supplement: Additional file 1 — Figure S1. Relationship between DNA methylation in regions of different distances to transcriptional start sites and gene expression levels in the human cerebral cortex. [file 1471-2148-12-144-S1.pdf]

Supplementary figures

Figure S1 Relationship between DNA methylation in regions of different distances to transcriptional start sites and gene expression levels in the human cerebral cortex.

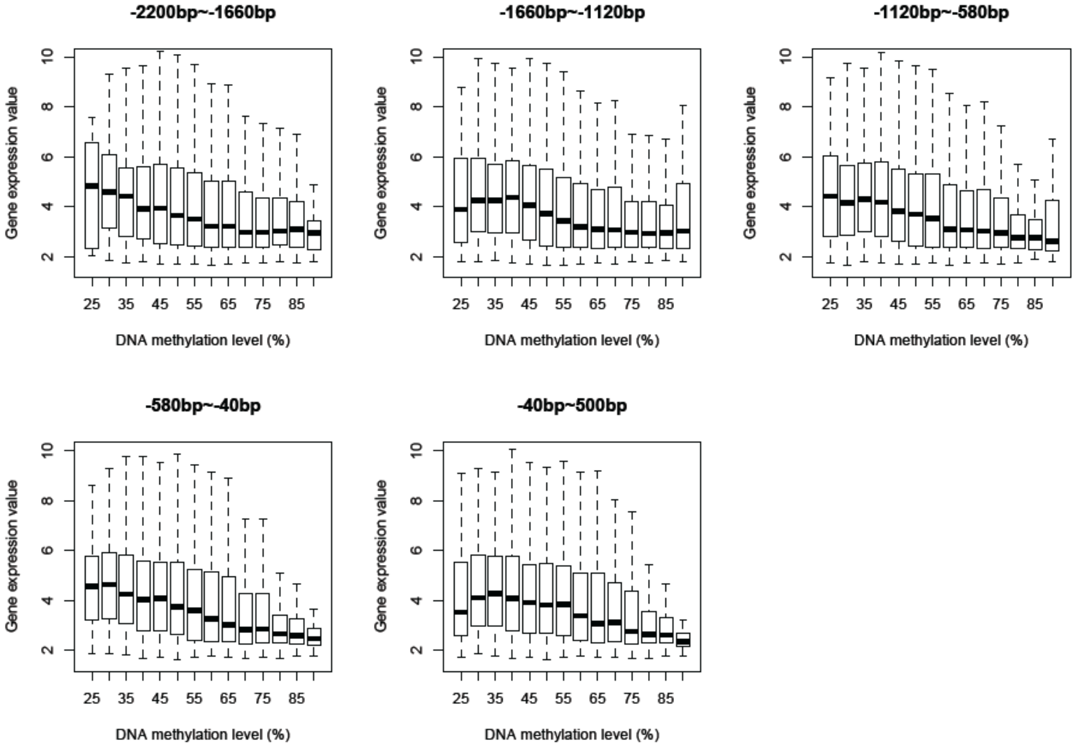

The 540bp regions were grouped into bins based on DNA methylation level, the label on x axis represent the minimum methylation level in the bin, for example, 25 represent the bin had methylation level range between 25% and 30%.
