# Supplementary material for: Genome-wide DNA methylation analyses in the brain reveal four differentially methylated regions between humans and non-human primates
Source: BMC Evol Biol. 2012 Aug 16;12:144. doi: 10.1186/1471-2148-12-144 (PMC3483258; doi:10.1186/1471-2148-12-144)
Supplement: Additional file 2 — Table S1. The numbers of DMRs identified by the Batman method when analyzing the 450 peaks identified in either of the two species by the NimbleScan method. [file 1471-2148-12-144-S2.doc]

**Table S1** The numbers of DMRs identified by the Batman method when analyzing the 450 peaks identified in either of the two species by the NimbleScan method.

| Total peaks | P<0.001 | P<0.01 | P<0.05 | P<0.1 |
| --- | --- | --- | --- | --- |
| 450 | 17 (3.8%) | 93 (20.7%) | 189 (42.0%) | 239 (53.1%) |

Total peaks: The total DNA methylation peaks that were found only in one species by NimbleScan.

The P value is the P value (t test) of different methylation between human and rhesus macaque using Batman method.

To get 150 candidate DMRs, the regions that showed large differences of CpG numbers between the two species were then manually removed from the 189 regions that were also significant (P<0.05) using Batman method.
